# Biochemical compounds and stress markers in lettuce upon exposure to pathogenic *Botrytis cinerea* and fungicides inhibiting oxidative phosphorylation

**DOI:** 10.1007/s00425-022-03838-x

**Published:** 2022-02-10

**Authors:** Piotr Iwaniuk, Bozena Lozowicka

**Affiliations:** Institute of Plant Protection-National Research Institute, Chelmonskiego 22 Street, 15-195 Bialystok, Poland

**Keywords:** Antioxidants, Azoxystrobin, Biochemistry, Dissipation, Fluazinam, Gray mould

## Abstract

**Main conclusion:**

*Botrytis cinerea* and fungicides interacted and influenced selected biochemical compounds. DPPH and glutathione are the first line of defence against biotic/abiotic stress. Plant metabolites are correlated with fungicides level during dissipation.

**Abstract:**

*Botrytis cinerea* is an etiological agent of gray mould in leafy vegetables and is combated by fungicides. Fluazinam and azoxystrobin are commonly used fungicides, which inhibit oxidative phosphorylation in fungi. In this study, lettuce was (i) inoculated with *B. cinerea*; (ii) sprayed with azoxystrobin or fluazinam; (iii) inoculated with *B. cinerea* and sprayed with fungicides. This investigation confirmed that *B. cinerea* and fungicides affected lettuce’s biochemistry and stress status. *B. cinerea* influenced the behaviour of fungicides reflected by shortened dissipation of azoxystrobin compared to non-inoculated plants, while prolonged degradation of fluazinam. Stress caused by *B. cinerea* combined with fungicides reduced level of chlorophylls (53.46%) and carotenoids (75.42%), whereas increased phenolic compounds (81%), ascorbate concentrations (32.4%), and catalase activity (116.1%). Abiotic stress caused by fungicides contributed most to the induction of carotenoids (107.68 µg g^−1^ on dissipation day 3^−1^). Diphenyl picrylhydrazyl (DPPH) radical scavenging activity and glutathione concentration peaked from the first hour of fungicides dissipation. For the first time correlation between the status of plant metabolites and fungicides during their dissipation was observed. These results indicate that non-enzymatic antioxidants could be the first-line compounds against stress factors, whereas ascorbate and antioxidant enzymes tend to mitigate stress only secondarily. The findings of this study help better understand plant biochemistry under biotic/abiotic stress conditions.

**Supplementary Information:**

The online version contains supplementary material available at 10.1007/s00425-022-03838-x.

## Introduction

Lettuce (*Lactuca sativa* L.) is a commonly consumed leafy vegetable, which is mostly cultivated in China, USA, and India. It is a rich source of fibre, minerals, and vitamins, such as mainly A, B, C, and E. High levels of antioxidant compounds, especially phenolic ones, determine how much they promote health. In addition, lettuce is a rich source of folic acid, lutein, and zeaxanthin, which protect against macular degeneration, as well as indoles with anti-cancer properties (Tahboub et al. 2010). Long-term intake has anti-inflammatory effects and actively reduces cholesterol.

One of the most common fungal diseases in lettuce is gray mould, which is caused by *Botrytis cinerea*. The fungus develops optimally at temperatures from 17 to 24 °C and a humidity of approx. 90%, with its occurrence being favoured by long-lasting rainfall (Ciliberti et al. 2015). Gray mould deteriorates the yield and quality of lettuce, but from the biochemical point of view, it can induce cascades of metabolic processes that are not fully understood yet. In plants infected by *B. cinerea*, Veillet et al. (2017) have demonstrated modulated sugar metabolism and enhanced uptake of hexoses. Moreover, some of the biochemical effects of *B. cinerea* depend on the host plant and its variety (Zhang et al. 2016). Plant tissues rich in antioxidants are less susceptible to *B. cinerea* infection, whereas H_2_O_2_ and superoxide dismutase are the main markers of biotic stress against this pathogen (Bui et al. 2019). Fungicides presenting a similar mechanism of action, mainly belonging to the group of strobilurins and carboxamides, are used for the protection of lettuce crops against gray mould.

Recent studies (Parween et al. 2014; Lozowicka et al. 2016; Wolejko et al. 2016) show that pesticides induce various changes in the metabolism of certain biochemical compounds. It has been observed that, among fungicides, flumioxazin, benlate, and calixin tend to reduce the concentration of carbohydrates and proteins in grapes and wheat (Siddiqui and Ahmed 2002; Saladin et al. 2003), whereas benzimidazole and dithiocarbamate decrease photosynthetic activity and promote the synthesis of carbohydrates (Singh and Sahota 2018). The use of pesticides can be considered an abiotic stress factor for plants, as, due to oxygen metabolism in the chloroplasts, mitochondria, cell membranes, and peroxisomes, it leads to the formation of reactive oxygen species (ROS). Fungicides such as tolclofos-methyl, flutolanil, and fludioxonil increase the activity of antioxidant enzymes in plants, which are of crucial importance to their response to environmental stress and the inhibition of ROS formation, although different antioxidant mechanisms may be involved in the detoxification of individual active ingredients (a.i.) (Mohamed and Akladious 2017). The antioxidant status of plants depends on the activity of their antioxidant enzymes (predominantly catalases, peroxidases, and dismutases) and non-enzymatic antioxidants (e.g., ascorbate, glutathione, and DPPH radical), which can have various effects on how the defence response against various types of pesticides proceeds. Furthermore, due to different modes of action, fungicides belonging to different chemical groups can affect the physiology of plants and the profile of biochemical compounds in various ways. In fungicides, the major modes of action are the inhibition of sterol biosynthesis and the disruption of respiration on multiple levels of oxidative phosphorylation (Yang et al. 2011). The dissipation rate of new fungicides in lettuce cultivation is desirable due to how the grace period is calculated. Although non-persistent pesticides tend to be employed, in particular in fruits and vegetables, their fate may differ. Research on the relationship between fungicide, fungus, and plant systems and their combined impact on plant biochemistry, in particular as regards the dissipation of pesticides over time intervals, is still insufficient.

Chemical protection of plants is crucial for obtaining healthy crops that show no symptoms of disease. However, the use of pesticides may induce disturbances in the concentration of biochemical compounds in plants, decreasing the proportion of nutrients that are desirable in terms of human consumption. Hence, there is a particular need for research into the prolonged impact of fungi and pesticides on the biochemistry of plants during the dissipation of fungicides. Accordingly, the purpose of this study was to determine how *B. cinerea* and fungicides with inhibition of oxidative phosphorylation (azoxystrobin, fluazinam) can affect the content of biochemical compounds and the incidence of stress markers in lettuce. Furthermore, a multifactorial relationship between the dissipation of fungicides, the content of biochemical compounds, and the incidence of stress markers could be demonstrated. We hypothesised that: (i) The dissipation rate of fungicides varies between non-inoculated lettuce and that inoculated with *B. cinerea*. (ii) The content of biochemical compounds and the incidence of stress markers differs in non-inoculated and inoculated lettuce and correlates with the concentration of residual fungicides. (iii) The behaviour of fungicides and their impact on plant biochemistry is similar between fungicides that inhibit oxidative phosphorylation.

## Materials and methods

### Chemicals and reagents

Fungicide analytical standards (azoxystrobin, fluazinam, purity > 98%) were purchased from Dr. Ehrenstorfer Laboratory (Augsburg, Germany). Analytical-grade solvents for chromatographic analysis (ammonium formate, acetonitrile) and formic acid were obtained from Sigma-Aldrich (Steinheim, Germany). QuEChERS sorbents (magnesium sulphate, sodium chloride, sodium citrate dihydrate, di-sodium hydrogen citrate 1.5-hydrate) and clean-up salts: graphitized carbon, primary-secondary amine, magnesium sulphate (GCB/PSA/MgSO_4_) were procured from UCT (Bristol, PA, USA). Pesticides with azoxystrobin (22.81%) and fluazinam (39.2%) were purchased from Syngenta (Basel, Switzerland) and Adama (Warsaw, Poland), respectively. Reagents for biochemical compounds and stress markers analysis (e.g., methanol, NaOH, sulphuric acid 95%, phenol solution 10%, ethanol, and Folin–Ciocalteu reagent) were obtained from Sigma-Aldrich.

### Experiment design

*Lactuca sativa* seeds were sown in the experimental pots with soil (10 cm of diameter, 1 seed per pot) in four repetitions to achieve repeatability, in phytotron (Pol-Eko KK 1450 TCP +). Soil compounds were as follows: 0.5 mg g^−1^ P_2_O_5_, 0.061 mg g^−1^ K_2_O, 0.084 mg g^−1^ Mg and pH 7.2. Pots were set at 30 cm interspace under controlled growth conditions of temperature (16/12 °C day/night) and photoperiod (12/12 h day/night). The average humidity was 80%. The plants were watered once a week with 200 mL of water per pot.

*Botrytis cinerea* was obtained from the Bank of Plant Pathogens of the Institute of Plant Protection-National Research Institute (Poznan, Poland) and was cultured in 0.5xPDB (Oxoid, Hampshire, UK) for 3 weeks at 14 °C. Culture was centrifuged at 11,000*g* for 5 min and spores in supernatant were adjusted to the optical density (OD) of 0.8 at a wavelength of 600 nm using Spectrophotometer Implen P300 (München, Germany). Lettuce was sprayed with spores’ suspension at BBCH 33. Azoxystrobin (182.48 mL ha^−1^) and fluazinam (156.8 mL ha^−1^) were applied at BBCH 37 (Table 1). Whole lettuce heads were collected after 1 h, 12 h, 1 day, 3 days, 5 days, 12 days, and 26 days after fungicides application, milled using a laboratory mill (Waring Commercial, Torrington, CT, USA), and stored at −20 °C until further use. Leaves for stress markers determination were stored at −80 °C and ground in the mortar just before analysis. All examined compounds were analysed in quadruplicates.

**Table 1:** Characteristics of fungicides used in this study

Active ingredient	Chemical group	Chemical structure	Mode of action	Molar mass (g M^−1^)	Log*P*	Solubility in water (mg L^−1^)
Azoxystrobin	Strobilurin		Complex III inhibition of fungal respiration	403.4	2.5	6.7
Fluazinam	Phenylpyridinamine		Uncoupler of oxidative phosphorylation	465.14	4.87	0.135

### Fungicide extraction and analysis by GC–MS/MS

**Fig. 1:**
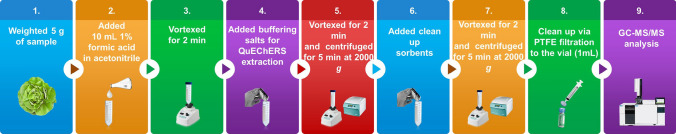
Detailed extraction and determination procedure of fungicides from lettuce

Fungicides were extracted from milled lettuce heads (5 g) using the QuEChERS method, based on the following buffering sorbents: 4 g MgSO_4_, 1 g NaCl, 1 g Na_3_C_6_H_5_O_7_ × 2H_2_O and 0.5 g Na_2_HC_6_H_5_O_7_ × 1.5H_2_O and clean-up sorbents: GCB/PSA/MgSO_4_ (2.5/25/150 mg), according to Wolejko et al. (2017) and Rutkowska et al. (2020). Full extraction and determination procedure are detailed in Fig. 1.

Gas chromatography coupled with tandem mass spectrophotometry (GC–MS/MS) was performed for azoxystrobin and fluazinam determination as previously described by Rutkowska et al. (2020). An Agilent 7890A GC system (Agilent Technologies, Palo Alto, CA, USA) was equipped with an Agilent 7693 autosampler coupled to a triple quadrupole mass spectrometer 7000B (Agilent Technologies). The device was operated in electron ionization mode (EI − 70 eV). Splitless injection sample (2 μL) was separated by an HP-5 MS capillary column [(5%-phenyl)-methylpolysiloxane; 30 m × 0.25 mm ID, 0.25 µm, Agilent Technologies]. The following oven temperature was used: 70 °C (2 min) to 150 °C at a rate 25 °C min^−1^, increased to 200 °C at 3 °C min^−1^ and finally to 280 °C at 8 °C min^−1^, held for 10 min. As the carrier gas helium (99.9% purity) was used (flow rate 2.1 mL min^−1^). The total running time was 41.88 min. The transfer line, ion source, and first and second quadrupole temperatures were as follows: 280, 300, 180, and 180 °C, respectively. As the collision gases, helium (99.9% purity, flow rate 2.25 mL min^−1^) and nitrogen (99.9% purity, flow rate 1.5 mL min^−1^) were used. MassHunter software (version B.06.00) (Agilent Technologies) was operated for quantitative analyses (Fig. S1 a, b).

### Validation of GC–MS/MS method

The validation of chromatographic method was conducted according to fungicide-free lettuce samples (SANTE 2019). The recovery, precision, linearity, matrix effect, limits of detection (LOD), and quantification (LOQ), were evaluated. Calibration curves of fungicide solutions in a lettuce blank matrix at final concentration 0.001, 0.01, 0.10, 0.50, 2.5, 5.0, 10.0 µg g^−1^ were prepared. Linearity was constructed based on the coefficients of determination (*R*^2^). Recovery data were determined in three concentration levels of the matrix to evaluate relative standard deviation (RSD). The LOQs with high accuracy and precision were indicated. LODs were established using three times signal-to-noise ratio (3 S/N). All details are listed in Table S1.

### Dissipation kinetics of fungicides under controlled conditions (dynamics)

First-order kinetics were calculated to determine pesticide concentration in time, according to Beulke and Brown (2001), and Lozowicka et al. (2017). They were indicated based on the equation: *C*_*t*_ = *C*_0_∙e^−*kt*^, where *C*_*t*_ is the concentration at the time of *t* (µg g^−1^); *C*_0_ is the concentration at the time zero *t* = 0 (µg g^−1^), initial deposits; *t* is the time; *k* is the constant degradation rate, in days. The half-life was indicated based on the *k* values of experiments *t*(1⁄2) = ln2/*k*.

### Determination of biochemical compounds

Chlorophyll *a*, *b* and carotenoids were extracted from milled lettuce heads (100 mg) with 5 mL of methanol according to Gu et al. (2016). Absorption was measured at wavelengths of 653 nm (chlorophyll *a*), 666 nm (chlorophyll *b*), and 470 nm (carotenoids) using Spectrophotometer Implen P300 (München, Germany).

Total soluble carbohydrates (TSC) were determined using modified phenol–sulphuric acid method (Jain et al. 2017). TSC were extracted from 100 mg of plant material in 5 mL 80% ethanol. A volume of 0.5 mL of 2% phenol solution was added to the extracts followed by 1.25 mL 96% sulphuric acid. TSC absorption was measured at the wavelength of 490 nm according to glucose/fructose/galactose (1:1:1, by vol.) calibration curve.

Protein was extracted from 100 mg of milled lettuce leaves with 2.5 mL 1 M NaOH. Protein concentration was assessed using Folin–Ciocalteu reagent according to Mæhre et al. (2018). Protein absorption was measured at the wavelength of 750 nm.

Phenolic compounds were extracted from 100 mg of milled plant material incubated for 1 h in 5 mL of distilled water at 40 °C. Phenolic compounds were determined according to Alvarez et al. (2016) at the wavelength of 765 nm according to gallic acid calibration curve.

### Determination of stress markers

For antioxidant enzymes’ activity determination, lettuce (0.5 g) was ground in chilled mortar with phosphate buffer pH 7 for catalase (CAT) and NADH-dependent peroxidase (POD) determination or pH 7.8 for superoxide dismutase (SOD) activity assessment. Samples were centrifuged for 15 min at 17,000*g*, 4 °C. Supernatant was used for enzyme activity determination at wavelengths of 240 nm (CAT), 340 nm (POD), and 560 nm (SOD) according to Lozowicka et al. (2021). Enzyme activity was expressed as U of enzyme mg^−1^ of protein.

DPPH scavenging activity, glutathione, and ascorbate content was determined as follows: milled lettuce (100 mg) was incubated with 5 mL of methanol for 30 min. Samples were centrifuged for 5 min at 2500*g*. For DPPH scavenging activity, extracts were mixed with 0.5 mM DPPH solution and 0.1 M sodium acetate buffer, pH 5.5. Samples were incubated for 30 min in the dark and the absorption was measured at the wavelength of 517 nm. DPPH scavenging activity was expressed as % of inhibition according to the equation: 100(*A*_0_ − *A*_s_)/*A*_0_, where *A*_0_ is absorption of DPPH solution and *A*_s_ is the absorption of sample (Barros et al. 2007).

For glutathione determination, extracts were mixed with reaction buffer containing 0.1 M Na-phosphate buffer, pH 7, and 1 mM EDTA. Next, samples were mixed with 0.4% DTNB. Absorption was measured at the wavelength of 412 nm according to l-glutathione calibration curve (Malar et al. 2014).

For ascorbate determination, extracts were mixed with 0.2 M phosphate buffer, pH 7.4, and 10 mM DTT. Next, 0.5% N-ethylmaleimide (NEM), 10% trichloroacetic acid, and 42% phosphorous acid were added and gently mixed. In the final step 4% 2,2′-dipiridil and 3% FeCl_3_ were added (Kampfenkel et al. 1995). Samples were incubated at 42 °C for 40 min. Absorption was measured at a wavelength of 525 nm according to l-ascorbic acid calibration curve.

### Statistical analysis

Statistical significance was calculated using post hoc Fischer’s test (*P* < 0.05). One-way ANOVA analysis of the effect of *B. cinerea* and fungicides on biochemical compounds and stress markers were conducted. Examined variables were correlated in PCA (Principal Component Analysis). Determined Pearson’s correlation coefficients between variables were visualized using heatmap. All data for statistical significance analysis were calculated in Statistica 12 software (StatSoft, Tulsa, OK, USA).

## Results

### Validation of GC–MS/MS

The method was validated in accordance with the guidelines for linearity, accuracy (expressed as recovery), inter- and intra-day precision, limit of quantification (LOQ), matrix effects, and uncertainty. For the concentration range under investigation (0.001–10.0 µg g^−1^), an optimum linearity of the method was obtained, with correlation coefficient *R*^2^ > 0.999. Limits of detection (LODs) for azoxystrobin and fluazinam were 0.001 and 0.003 µg g^−1^, respectively. For azoxystrobin and fluazinam, LOQs were quantified with acceptable accuracy (recovery > 70%) and precision (RSD < 20%) levels at 0.005 µg g^−1^. With the method applied, average recovery rates for azoxystrobin and fluazinam amounted to 88% and 76%, respectively. Ranging from 19% to 20%, the matrix effect had no effect upon signal attenuation or amplification (Table S1).

### Dissipation of fungicides in non-inoculated lettuce and that inoculated with *B. cinerea*

**Fig. 2:**
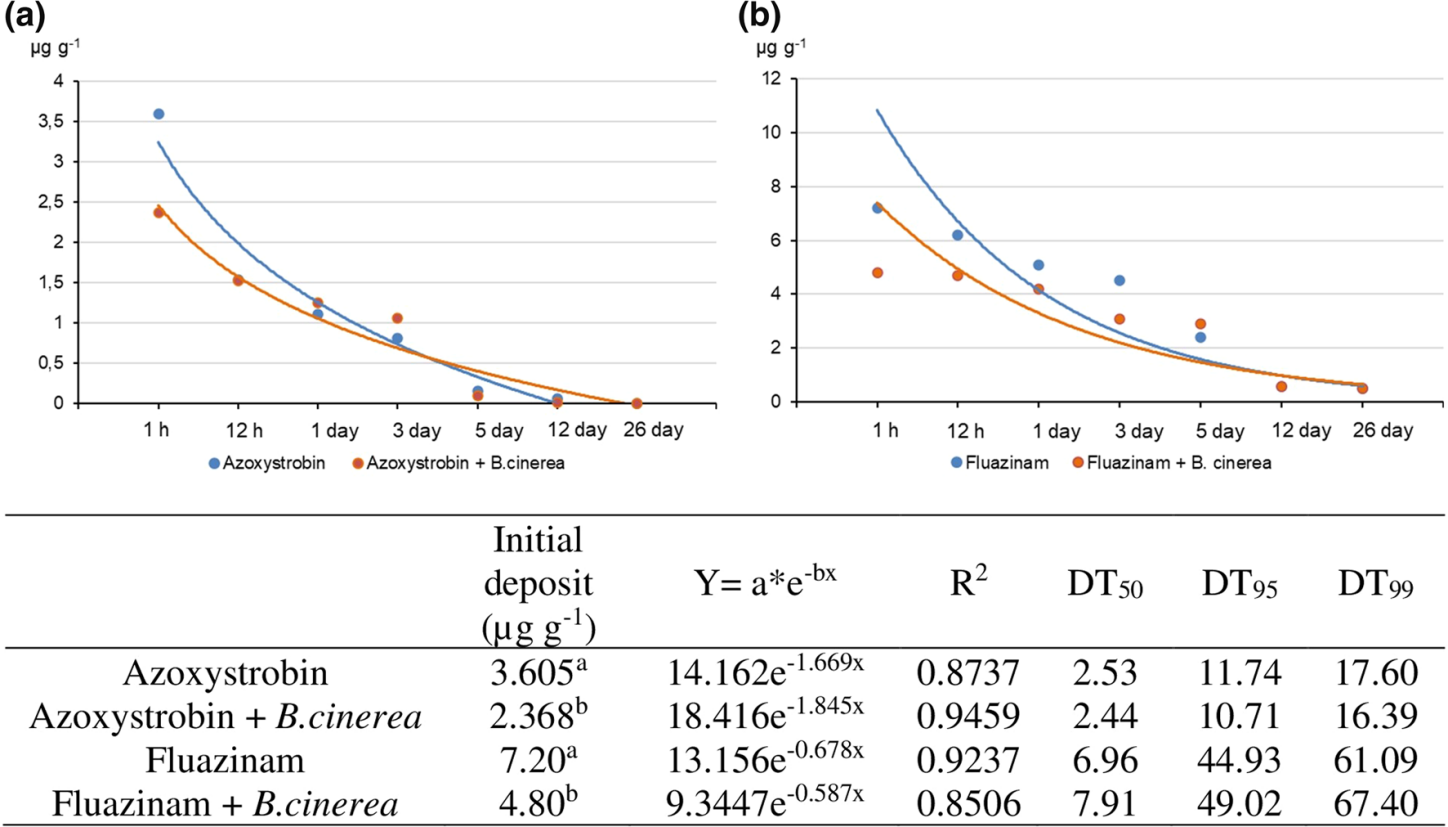
Dissipation kinetics, initial deposit, equation of dissipation, and degradation time (50%, 95%, and 99%) of fungicides inhibiting oxidative phosphorylation in non-inoculated lettuce and that inoculated with *B. cinerea*. **a** Azoxystrobin. **b** Fluazinam (*P* < 0.05, *n* = 4)

The degradation times of azoxystrobin and fluazinam in healthy lettuce and that inoculated with *B. cinerea* are shown in Table 2. Following the first-order kinetic equation (*C*_*t*_ = *C*_0_∙e^−*kt*^), the concentration of fungicides decreased at different rates. Determination coefficients were within an acceptable range (*R*^2^ = 0.8506–0.9459). Higher initial deposits could be identified in non-inoculated samples (3.605 µg g^−1^ for azoxystrobin and 7.20 µg g^−1^ for fluazinam). The shortest half-life in non-inoculated lettuce was determined for azoxystrobin (DT_50_ = 2.53) (Fig. 2a), whereas fluazinam needed more time to be degraded (DT_50_ = 6.96) (Fig. 2b). The presence of *B. cinerea* sped up the degradation of azoxystrobin (by 3%), whereas the dissipation of fluazinam was more prolonged (Fig. 2).

**Table 2:** Accumulation of fungicides in non-inoculated lettuce and that inoculated with *B. cinerea*

Period	Non-inoculated		Inoculated	
	Concentration (µg g^−1^)	Loss (%)	Concentration (µg g^−1^)	Loss (%)
Azoxystrobin				
1 h	3.605	–	2.368	–
12 h	1.542	57.2	1.52	35.8
1 day	1.109	69.2	1.254	47.0
3 days	0.815	77.4	1.064	55.1
5 days	0.15	95.8	0.093	96.1
12 days	0.057	98.4	0.016	99.3
26 days	0.001	99.9	0.001	99.9
Fluazinam				
1 h	7.20	–	4.80	–
12 h	6.20	13.9	4.70	2.1
1 day	5.10	29.2	4.20	12.5
3 days	4.50	37.5	3.10	35.4
5 days	2.40	66.7	2.90	39.6
12 days	0.60	91.7	0.60	87.5
26 days	0.50	93.1	0.50	89.6

### Concentration of biochemical compounds during the degradation of fungicides in non-inoculated lettuce and that inoculated with* B. cinerea*

**Fig. 3:**
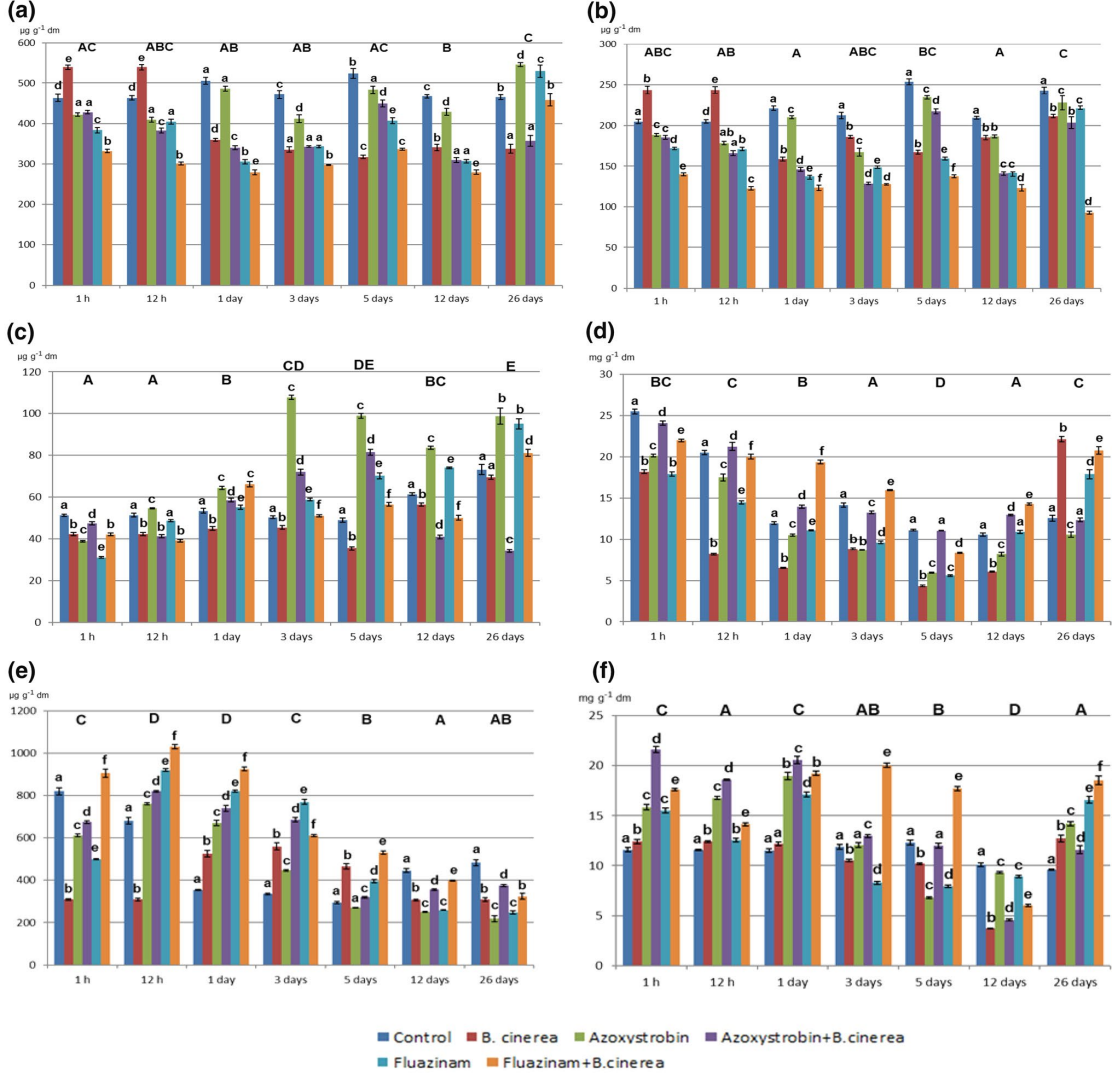
Concentration of biochemical compounds during the degradation of fungicides in non-inoculated lettuce and that inoculated with *B. cinerea*. **a** Chlorophyll a. **b** Chlorophyll b. **c** Carotenoids. **d** Total carbohydrates. **e** Phenolic compounds. **f** Protein. The same lower case letters among the time of dissipation indicate not significant difference (*P* > 0.05) between treatments (*n* = 4). The same upper case letters indicate not significant difference (*P* > 0.05, *n* = 4) between fungicides concentration (time of dissipation)

The presence of *B. cinerea* and fungicides had an impact on the concentration of chlorophylls, carotenoids, total carbohydrates, protein, and phenolic compounds in lettuce. *B. cinerea* combined with fungicides caused a more pronounced decrease in the concentration of chlorophyll a, chlorophyll b and carotenoids compared to non-inoculated treatments (up to 43.97% and 75.42% for chlorophyll a and carotenoids on the first and 26th day of treatment with azoxystrobin, and up to 57.97% for chlorophyll b during treatment with fluazinam) (Fig. 3a–c). Despite the dissipation of fungicides, the concentration of chlorophylls in most plants under study was lower compared to control lettuce. The use of fungicides in non-inoculated samples was responsible for the accumulation of carotenoids from the third day (Fig. 3c). Basically, lower concentrations of chlorophyll *a*, *b* and carotenoids were determined in inoculated plants treated with fluazinam (278.9, 123.02, 39.12 µg g^−1^, respectively).

Inoculation with *B. cinerea* caused an increase in total carbohydrates compared to non-inoculated lettuce (up to 38.02% for fluazinam, on the first day). Starting from the first hour after the use of pesticides, the total concentration of carbohydrates gradually decreased, reaching its minimum level (5.35–11.05 mg g^−1^) for inoculated and non-inoculated plants on the fifth day and starting to rise again from the 12 day on. The highest concentration of carbohydrates was determined for azoxystrobin (24.1 mg g^−1^) and for fluazinam (22 mg g^−1^) combined with *B. cinerea* 1 h after the use of pesticides (Fig. 3d).

However, the highest phenolic compounds’ concentrations were observed in inoculated lettuce (an up to 44.75% increase on the first hour for fluazinam). The mere use of fungicides caused significant accumulation of phenolic compounds for azoxystrobin (760 µg g^−1^) and for fluazinam (920 µg g^−1^) after 12 h (Fig. 3e). The proportion of phenolic compounds was relatively high in both inoculated and non-inoculated samples until the third day (1030 µg g^−1 ^for fluazinam).

Compared to non-inoculated plants, inoculation with *B. cinerea* resulted in a higher concentration of protein (up to 21.61 mg g^−1^ after 1 h). A major reduction in protein concentration was determined on the fifth and the 12 days (up to 4.55 mg g^−1^ for azoxystrobin combined with *B. cinerea*) (Fig. 3f).

### Activity of antioxidant enzymes and the concentration of non-enzymatic antioxidants during the degradation of fungicides in non-inoculated lettuce and that inoculated with* B. cinerea*

**Fig. 4:**
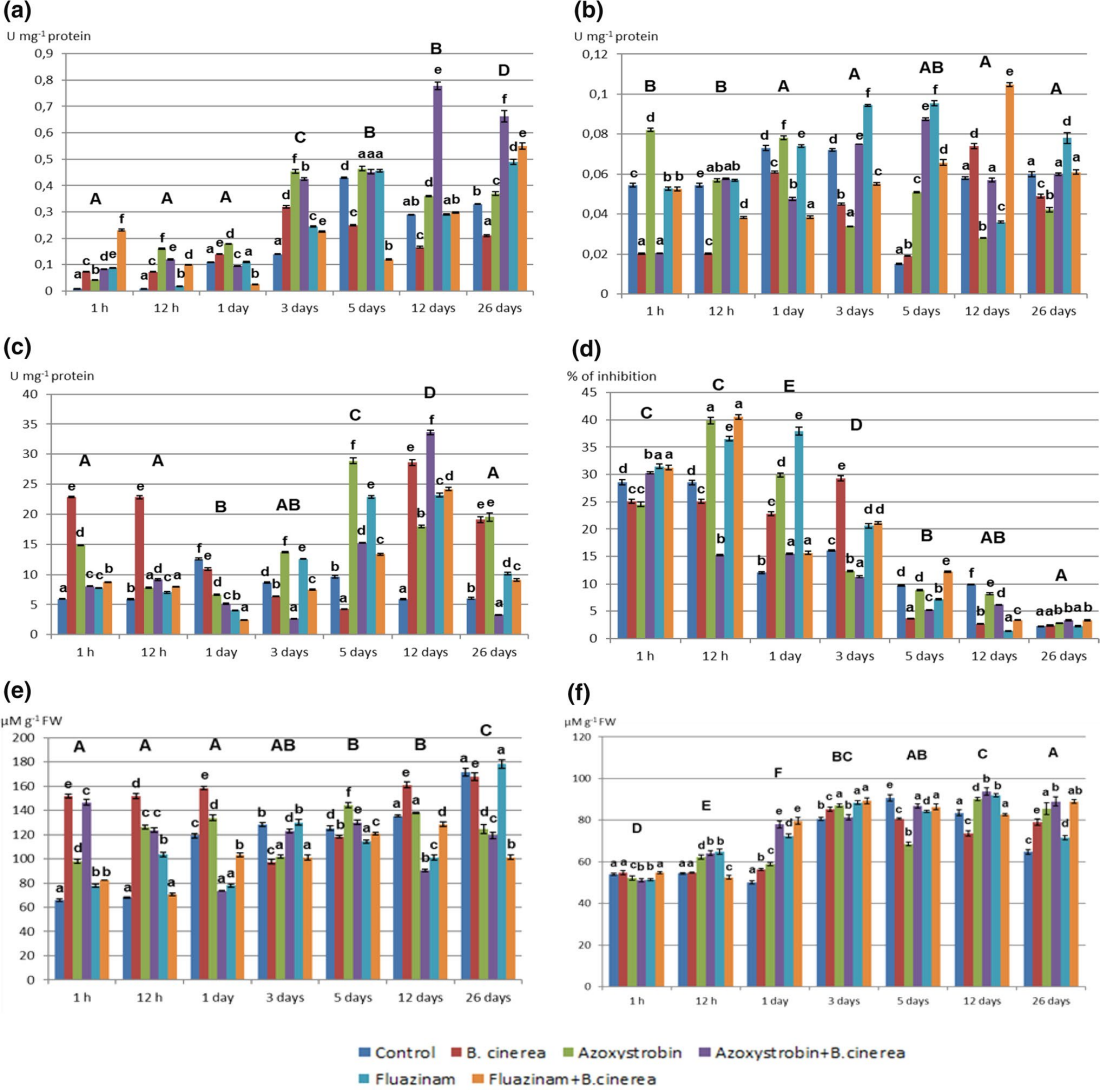
Activity of antioxidant enzymes and concentration of non-enzymatic antioxidants during the degradation of fungicides in non-inoculated lettuce and that inoculated with *B. cinerea*. **a** CAT activity. **b** NADH-dependent POD activity. **c** SOD activity. **d** DPPH radical scavenging activity. **e** Glutathione concentration. **f** Ascorbate concentration. The same lower case letters among the time of dissipation indicate not significant difference (*P* > 0.05) between treatments (*n* = 4). The same upper case letters indicate not significant difference (*P* > 0.05, *n* = 4) between fungicides’ concentration (time of dissipation)

*B. cinerea* was indicated as a biotic stress factor for this study, with the use of fungicides as an abiotic stress being capable of considerably influencing on the antioxidant profile of lettuce depending on how pesticides dissipate (Fig. 4). Compared to non-inoculated lettuce, *B. cinerea* contributed to a higher CAT, POD, and SOD activity in particular time periods, intensifying the antioxidant response when several stress factors overlapped. Compared to inoculation with *B. cinerea* alone, the steepest rise in the activity of antioxidant enzymes in inoculated lettuce could be observed for azoxystrobin on the twelfth (CAT: 78.62% increase) (Fig. 4a) and the fifth days (POD: 78.2%; SOD: 72.07%) (Fig. 4b, c), whereas for fluazinam, it was on the 26th (CAT: 61.1%) and the fifth days (POD: 70.2%; SOD: 68.09%). In addition, the CAT activity gradually increased, reaching for azoxystrobin and fluazinam its maximum levels in non-inoculated samples between the fifth and the 26th days after the use of fungicides (0.46 and 0.49 U mg^−1^, respectively). The POD activity in non-inoculated plants was the highest until the fifth day (0.082 and 0.095 U mg^−1^ for azoxystrobin and fluazinam, respectively). The SOD activity in non-inoculated plants reached its maximum level between the fifth and the twelfth days (28.89 and 23.197 U mg^−1^ for azoxystrobin and fluazinam, respectively) (Fig. 4c).

For azoxystrobin, and partially also for fluazinam, an increased antioxidant capacity of DPPH radical and a higher concentration of glutathione were determined in non-inoculated samples. The highest DPPH radical scavenging activity could be observed in most plants until three days after the use of fungicides (for azoxystrobin after 12 h, 39.82% and for fluazinam on the first day, 37.99%) (Fig. 4d), whereas the greatest concentration of glutathione was determined for azoxystrobin (144.11 µM g^−1^) on the fifth day and for fluazinam (178.26 µM g^−1^) on the 26th day (Fig. 4e). In contrast to DPPH radical and glutathione, the concentration of ascorbate was generally higher in inoculated lettuce (24.49% increase for azoxystrobin on the first day and 19.56% increase for fluazinam on 26th day as compared to non-inoculated plants). Starting from the first day after the use of fungicides, the concentration of ascorbate gradually increased. The highest level in non-inoculated samples was determined for azoxystrobin (90.15 µM g^−1^) and fluazinam (91.9 µM g^−1^) on the twelfth day (Fig. 4f).

### Chemometric analysis

**Fig. 5:**
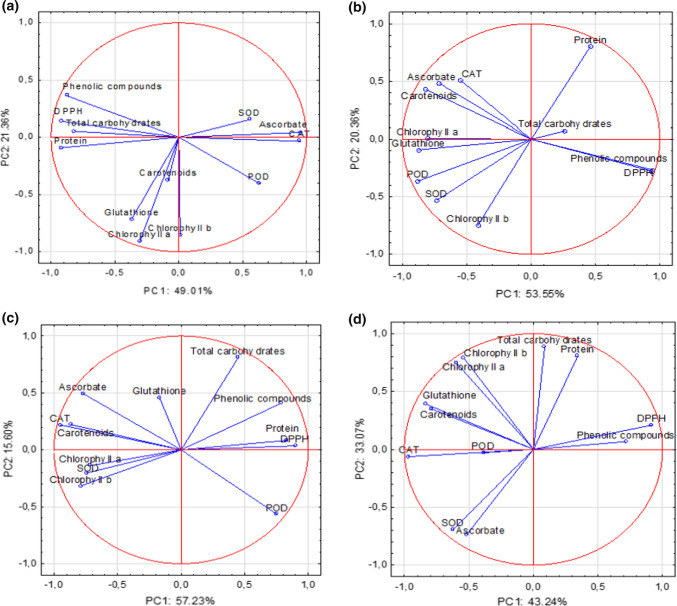
Principal component analysis of the fungicides and *B. cinerea* impact on biochemical compounds and stress markers in lettuce (*P* < 0.05, *n* = 4). **a** Azoxystrobin + *B. cinerea*. **b** Fluazinam + *B. cinerea*. **c** Azoxystrobin. **d** Fluazinam

**Fig. 6:**
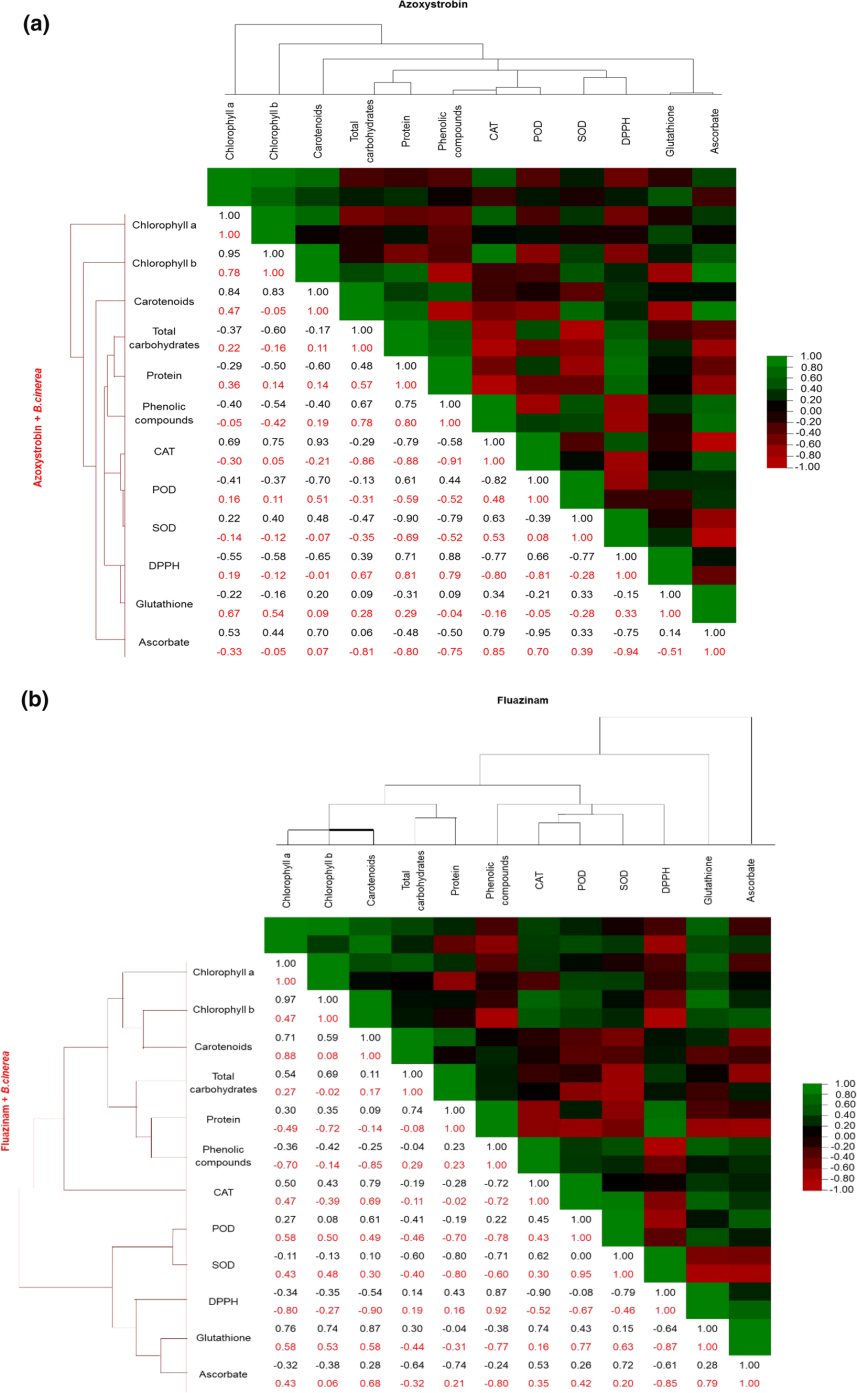
Heatmap based on Pearson’s correlation coefficients of the fungicides and *B. cinerea* impact on biochemical compounds and stress markers in lettuce (*P* < 0.05, *n* = 4). **a** Azoxystrobin. **b** Fluazinam

The significance of the findings was confirmed by statistical analysis. The total variability in inoculated plants between the individual parameters under study and the dissipation of fungicides was confirmed by 70.37% (PC1 = 49.01%, PC2 = 21.36%) for azoxystrobin and 73.91% (PC1 = 53.55%, PC2 = 20.36%) for fluazinam (Fig. 5a, b). It demonstrated that, despite its prolonged degradation by *B. cinerea*, fluazinam has a greater impact  on biochemical compounds and stress markers. In addition, how the dissipation of azoxystrobin and fluazinam in non-inoculated lettuce affects biochemical compounds and stress markers in particular time points could be explained with a total variability of 72.83% (PC1 = 57.23%, PC2 = 15.60%) and 76.31% (PC1 = 43.24%, PC2 = 33.07%), respectively (Fig. 5c, d). Furthermore, the highest positive correlations in treatment with azoxystrobin were indicated by the findings of the study between chlorophyll *a* and chlorophyll *b* (*r* = 0.95), between chlorophyll *a* and carotenoids (*r* = 0.84), between phenolic compounds and protein (*r* = 0.75), and between phenolic compounds and total carbohydrates (*r* = 0.67) (Fig. 6a). Among stress markers, the strongest relation was found between CAT and carotenoids (*r* = 0.93), between CAT and ascorbate (*r* = 0.79), between POD and protein (*r* = 0.61), and between DPPH and phenolic compounds (*r* = 0.88). For treatment with azoxystrobin, *B. cinerea* inoculation resulted in lower correlations, especially between photosynthetic pigments and CAT (*r* = −0.30), whereas the closest ones could be identified between phenolic compounds and protein (*r* = 0.80) and between phenolic compounds and DPPH (*r* = 0.81) (Fig. 6a). By the same token, the closest correlations in treatment with fluazinam were those between chlorophyll *a* and chlorophyll *b* (*r* = 0.97), between chlorophyll *a* and carotenoids (*r* = 0.71), and between chlorophyll *a* and glutathione (*r* = 0.76) (Fig. 6b). Among antioxidants, higher correlations were determined for CAT and carotenoids (*r* = 0.79), for POD and carotenoids (*r* = 0.61), for glutathione and carotenoids (*r* = 0.87), and for CAT and SOD (*r* = 0.62). Interestingly, fluazinam combined with *B. cinerea* significantly reduced the correlation between protein and photosynthetic pigments (*r* = −0.72), between CAT and chlorophyll *b* (*r* = −0.39), and between DPPH and carotenoids (*r* = −0.90) and glutathione (*r* = −0.87) (Fig. 6b).

## Discussion

### Dissipation of fungicides in non-inoculated lettuce and that inoculated with* B. cinerea*

There were previous reports that many saprophytic microorganisms (e.g., *Bacillus, Pseudomonas, Saccharomyces*) can speed up the dissipation time of certain pesticides (e.g., glyphosate, MCPA, or sulfosulfuron) (Kaczynski et al. 2020; Lozowicka et al. 2021). Our study shows, however, that pathogenic fungi are also capable of metabolising selected fungicides, thus contributing to their faster degradation in plants. Azoxystrobin can be degraded by such fungi despite inhibiting action on oxidative phosphorylation in *B. cinerea*. Prolonged degradation of fluazinam is attributable to its low solubility in water solutions (0.135 mg L^−1^), compared to azoxystrobin, which degrades more rapidly (6.7 mg L^−1^) (Table 1). Accordingly, fluazinam cannot dissolve or be metabolised in the aqueous environment of fungal cell cytosol. In addition, the octanol–water partition and logP coefficients are the highest for fluazinam (Table 1) and can affect how it can be metabolised by fungi (Verma et al. 2014). As reflected by differences between DT_50_ in our results and average values in the PPDB database (DT_50_ = 4.2 for azoxystrobin and DT_50_ = 4.3 for fluazinam), it can also be assumed that the dissipation time of fungicides depends on plant species or organs.

### Concentration of biochemical compounds during the degradation of fungicides in non-inoculated lettuce and that inoculated with* B. cinerea*

Inoculation with *B. cinerea* and the use of fungicides triggered various temporary changes in lettuce’s biochemical compounds. The effects of pesticides on the concentration of photosynthetic pigments remain unclear. Chen et al. (2015) demonstrated that the concentration of chlorophyll in wheat leaves is lower after infection with the leaf pathogen *Puccinia striiformis*. There are reports that the use of malathion, thiamethoxam, tolclofos-methyl, and chlorpyrifos can increase pigment levels (Salem 2016). In addition, higher concentrations of photosynthetic pigments in response to heavy metals could be observed (Yang et al. 2020). By blocking the shikimate pathway and the biosynthesis of pigments, glyphosate can slightly reduce the concentration of carotenoids in *Medicago sativa*, however (Fernandes et al. 2020). Lower photosynthetic activity may also be attributable to some adverse effects of fungicides inhibiting oxidative phosphorylation on the   Rubisco enzyme activity. The use of pesticides seems to have some impact upon the concentration of pigments, depending on the mode of action of pesticides, their physicochemical properties, and metabolic pathways in plants. The use of fungicides combined with *B. cinerea* inoculation only intensified the effect of chlorophylls reduction due to the inhibition of photosynthetic enzymes or the disruption of photosystem I and photosystem II (Tung et al. 2013).

Interestingly, lower proportions of carbohydrates are most probably attributable to their involvement in the biosynthesis of PAMP, DAMP, and MAMP sugar-like compounds, which present a defence mechanism against pathogens (Trouvelot et al. 2014). In contrast to Schoneberg et al. (2018), the concentration of phenolic compounds was relatively high in both inoculated and non-inoculated samples until the third day. Biotic stress conditions seem to be more effective in inducing the biosynthesis of phenolic compounds. Phenolic compounds are antioxidants whose higher concentrations under stress conditions mitigate any unfavourable effects of the environment. Stress conditions can induce the formation of enzymes in the phenolic biosynthesis pathway (e.g., chalcone synthase) (Sharma et al. 2016). Phenolic compounds can mitigate negative effects of pollutants in plants, including pesticides, and inhibit the catalytic activity of protein kinases during ROS formation, which distorts the functions of nucleic acids, proteins, and lipids.

Furthermore, higher concentrations of protein in lettuce until the third day of treatments could be due to the biosynthesis of defence proteins and kinases, which distort the formation of ROS (Iwaniuk et al. 2022). In addition, a decrease in the concentration of protein after the fifth day implies that their biosynthesis is inhibited, probably by fungicides binding to the large ribosomal subunit or by protein degradation (Siddiqui and Ahmed 2002). The protein-related plant response after the use of pesticides requires more in-depth research including proteomic profile analysis.

### Activity of antioxidant enzymes and concentration of non-enzymatic antioxidants during the degradation of fungicides in non-inoculated lettuce and that inoculated with* B. cinerea*

Inoculation with *B. cinerea* and the use of fungicides triggered various temporary changes in lettuce’s profile of antioxidants. There are numerous reports showing a higher activity of antioxidant enzymes under stress conditions in one time point (Grigoryuk et al. 2016; Singh and Prasad 2018; Lozowicka et al. 2021). Our findings show, however, that higher activity of antioxidant enzymes is not constant during the dissipation of fungicides. Yang et al. (2019) demonstrated increased catalase activity during the dissipation of thidiazuronon in strawberries, whereas increased activity of peroxidase under biotic stress conditions may be the result of cell wall lignification (Debona et al. 2012). In contrast to increased POD activity after the use of imidacloprid (Sharma et al. 2017), the present study shows a moderate defence role of POD from the very beginning of biotic (*B. cinerea*) and abiotic stress (fungicides used to inhibit oxidative phosphorylation). Moreover, as reported by Shakir et al. (2018), antioxidant enzyme levels may vary depending on plant organs. CAT and SOD reached their maximum levels halfway during the biotic/abiotic stress induction phase. Accordingly, they are not the first line of antioxidant defence. Stajner et al. (2003) determined a lower SOD activity in lettuce treated with alachlor and metalachlor, whereas Yang et al. (2019) could observe a relatively high SOD activity during the dissipation of thidiazuron. This shows that the effects of pesticides on antioxidant enzymes depend on an active ingredient’s mode of action, plant species, and type of plant organs.

Our results show that DPPH radical activity is the highest at the beginning of biotic/abiotic stress occurrence. Krzepilko and Zych-Wezyk (2010) found DPPH radical activity under stress conditions to be greater when caused by lambda-cyhalothrin, which can form ROS. Next, DPPH couples unpaired electrons or hydrogen radicals. Moreover, glutathione is recognised as a strong antioxidant against biotic stress conditions (Zechmann 2020). Its capacity can be inhibited by certain pesticides, however. Hence, when combined with fungicides, its levels in inoculated lettuce are lower. Studies showing the glutathione behaviour during the dissipation of pesticides are scarce. Wang et al. (2010) found a higher glutathione concentration after 24 h from the use of chlorothalonil, whereas treatment with carbendazim ensured steady glutathione levels during the 96 h of the experiment. Compared to control, the antioxidant activity of glutathione is most pronounced at early stages of stress occurrence, which enables sequestration of pollutants (Gullner et al. 2018). Furthermore, ascorbate concentration levels rose from halfway through stress induction, whereas inoculation with *B. cinerea* intensified the accumulation of ascorbate levels. This was probably due to the induction of the common glutathione-ascorbate catabolism pathway (Kuzniak and Sklodowska 2005). In addition, De Sousa et al. (2017) could observe varying tendencies to accumulate ascorbate depending on leaves and roots.

## Conclusions

*B. cinerea* is an etiological agent of gray mould, but it also affects primary metabolites and stress markers in lettuce. Our findings show that, except for saprophytic microorganisms, *B. cinerea* can also shorten the degradation of fungicides. However, pesticides with low solubility in the aqueous environment of cytosol (e.g., fluazinam) are not degraded by microorganisms any faster. *B. cinerea* and fungicides that inhibit oxidative phosphorylation have been demonstrated to have various effects on plant biochemistry at various stages of dissipation, depending on the chemical group and physicochemical properties of fungicides. They contribute to the induction of carotenoids (halfway during and towards the end of fungicide dissipation) and the reduction of chlorophylls. Among nutrients, phenolic compounds and proteins present the first line of defence against biotic and abiotic stress. In addition, DPPH radical and glutathione are most involved at the onset of plant response. Therefore, non-enzymatic antioxidants can play a key role in the first line of defence against stress factors, whereas ascorbate and antioxidant enzymes support stress mitigation only secondarily. Furthermore, we concluded that the occurrence of additional biotic stress intensified the decrease of chlorophylls, whereas the growth of carbohydrates, phenolic compounds, the concentration of ascorbate, and the CAT and POD activity. For the first time, we could demonstrate that the profile of a plant’s biochemical compounds and stress markers is correlated with the amount of fungicides during their degradation. The findings of the study contribute to a better understanding of plant biochemistry under biotic/abiotic stress conditions.

### *Author contribution statement*

PI designed experiment, performed analysis, and wrote the manuscript. BL designed experiment and revised the final version of the manuscript. All authors read and approved the manuscript.

## Supplementary Information

Below is the link to the electronic supplementary material.

Fig. S1 Example of azoxystrobin chromatograms. **a** Standard (0.01 µg g^−1^).** b** Sample. Supplementary file1 (XLSX 172 KB)

Supplementary file2 (DOCX 24 KB)

## Data Availability

All data generated or analysed during this study are included in this published article [and its supplementary information files].

## Abbreviations

DT_50_: Degradation time (50%)
DPPH: Diphenyl picrylhydrazyl
CAT: Catalase
POD: NADH-dependent peroxidase
ROS: Reactive oxygen species
SOD: Superoxide dismutase
