# Supplementary material for: Biochemical compounds and stress markers in lettuce upon exposure to pathogenic Botrytis cinerea and fungicides inhibiting oxidative phosphorylation
Source: Planta. 2022 Feb 10;255(3):61. doi: 10.1007/s00425-022-03838-x (PMC8828598; doi:10.1007/s00425-022-03838-x)
Supplement: Supplementary file 2 — Supplementary file2 (DOCX 24 KB) [file 425_2022_3838_MOESM2_ESM.docx]

**Supplementary material**

**Table S1** Chromatographic parameters of the analysed fungicides

| Active ingredient | Matrix effect | Mean recovery  (%) | LOQ  (µg g^-1^) | LOD  (µg g^-1^) | Quantification | | Confirmation | | DP^b^ (V) | EP^c^ (V) |
| --- | --- | --- | --- | --- | --- | --- | --- | --- | --- | --- |
|  |  |  |  |  | MRM transition *m/z* | CE^a^ (V) | MRM transition *m/z* | CE^a^ (V) |  |  |
| Azoxystrobin | 19% | 88 | 0.005 | 0.001 | 344>329 | 15 | 344>172 | 40 | 344>183 | 25 |
| Fluazinam | 20% | 76 | 0.005 | 0.003 | 420>375 | 20 | 418>373 | 20 | 420>385 | 30 |

^a^ CE: Collision energy; ^b^ DP: Declustering potential; ^c^ EP: Collision chamber inlet voltage
